# Research and Remedies

**Published:** 1912-02-03

**Authors:** 


### RESEARCH AND REMEDIES.

### Ether Glycosuria.

Three papers have recently appeared on this subject, which was first ventilated by Ruschhaupt about twelve years ago. Thus King and three other authors detail in the *Bulletin of the Johns Hopkins Hospital* a long series of experiments, as the result of which they feel able to formulate certain definite conclusions, as follows: After the administration of ether to the point of deep anæsthesia, there results in healthy adult dogs a well-marked glycosuria. The reducing substance thus produced in the urine is dextrose; and the cause of the glycosuria is a definite hyperglycæmia. The liver is the organ which furnishes the increase of sugar to the blood, as shown by the absence of any hyperglycæmia and glycosuria when the liver is removed from the circulation by an Eck fistula. Severance of all the splanchnic fibres of the cœliac plexus going to the liver does not remove the hyperglycæmia and glycosuria set up by the ether.

The other two researches are published by Hawk in the *Archives of Internal Medicine.* This author concludes that the type of glycosuria in question is due primarily to the effect of ether in stimulating the transformation of glycogen into dextrose. He also finds that there is an inhibition of the secretion of urine during the administration of the anæsthetic, but this is quickly followed by hypersecretion which begins when the anæsthesia terminates. This inhibition is thought to be due to the constriction of the arterioles of the kidney by the direct action of ether on them. How far these conclusions are valid in the case of man is doubtful, and might profitably engage the attention of those who have done their experimental work on dogs.

### The Origin of Cerebro-Spinal Fluid.

Within the last decade physicians have paid much attention to the cerebro-spinal fluid as it is found around the spinal cord. They have tapped it by trocar and canula inserted between consecutive vertebræ, both for the relief of symptoms (as in uræmia, cerebral tumour, etc.); for diagnostic purposes (as in meningitis of various kinds, trypanosomiasis, and other infections); and as part of a therapeutic process (as in spinal anæsthesia). Yet little is certain about the formation of this fluid, which has always been supposed to be formed chiefly by the choroid plexus. In *Brain* an American investigator, Dr. Kramer, details some observations upon the function of this plexus and upon its share in the production of the cerebro-spinal fluid. He agrees with the old opinion that the choroid plexus is very largely responsible for the formation of normal cerebral fluid; and he goes further by suggesting that it is also capable of forming substances which have a depressant effect upon the blood pressure.

Evidence was obtained that this substance is of the globulin class, and experiments were then made to ascertain whether it is formed in excess when there is hypersecretion of cerebro-spinal fluid. Cerebro-spinal fluid from patients suffering from cerebral œdema due to injury, and from delirium tremens, caused intense depressor effects in dogs; whereas when the recovery stage from delirium *e potu* was reached, the cerebro-spinal fluid had practically no effect. In diabetic coma the same toxicity appears in the cerebro-spinal fluid. What bearing these interesting experiments may have on treatment can only be shown by further research; but it is evident that extensive possibilities are foreshadowed by these interesting observations.

### Tests for Melæna.

The great increase in the precision with which the diagnosis of gastric and duodenal ulceration can now be made, owing to the writings and observations of several authors, of whom Moynihan is the most prominent, has directed attention to the question of occult melæna. This valuable sign has become of great clinical importance, and hitherto the detection of it has been left mainly in the hands of expert pathologists. Colwell and MacCormac publish in the *Archives of the Middlesex Hospital* an improved technique, the chief merits of which are claimed to be that it is accurate and easy to carry out. Their directions read as follows: Dissolve 0.1 gm. of benzidine and 0.1 gm. of sodium perborate in 10 c.c. of glacial acetic acid: solution may be assisting by gentle stirring with a glass rod. The solution has at first a green colour, which quickly changes to a pale brown; the latter then slowly darkens. This solution should be freshly prepared for each case. If the suspect material is liquid (urine, vomit, or fæces) take 5 c.c. of it, and add 5 c.c. of glacial acetic acid: shake thoroughly in a stoppered 25 c.c. measure. Add 10 c.c of ether and shake again. Pipette off the supernatant ethereal extract.

If the material be solid fæces, rub up a piece the size of a pea with 5 c.c. of glacial acid, add 5 c.c. of water, and extract with ether as before. In either case, place 5 c.c. of the ethereal extract in a white evaporating dish. Encourage the ether to evaporate by the warmth of the hand and by gently blowing the surface. When the ether is all gone there will still be a strong smell of acetic acid. Then add 1 c.c. of the benzidine-perborate solution, and if blood was present in the original material an intense blue colour appears. Occasionally a green colour is seen; this will change to blue if another 1 c.c. of benzidine-perborate solution is added. The only fallacy in the test is that green vegetables and fresh meat will invalidate it. Therefore the patient must be placed for three days upon a diet from which these are excluded. Milk, eggs, tea, bread, butter, potatoes, meat extract, are all admissible. It is said that this test will detect one part in a million of blood. The presence of sugar or of ammoniacal compounds in the urine does not invalidate the reaction.

